# Mechanical Composite of LiNi_0.8_Co_0.15_Al_0.05_O_2_/Carbon Nanotubes with Enhanced Electrochemical Performance for Lithium-Ion Batteries

**DOI:** 10.1186/s11671-017-2143-4

**Published:** 2017-05-30

**Authors:** Liping Zhang, Ju Fu, Chuhong Zhang

**Affiliations:** State Key Laboratory of Polymer Materials Engineering, Polymer Research Institute, Sichuan University, Chengdu, 610065 China

**Keywords:** LiNi_0.8_Co_0.15_Al_0.05_O_2_ (NCA), Carbon Nanotubes (CNTs), Solid-State Grinding, Lithium-Ion Battery, Cathode

## Abstract

LiNi_0.8_Co_0.15_Al_0.05_O_2_/carbon nanotube (NCA/CNT) composite cathode materials are prepared by a facile mechanical grinding method, without damage to the crystal structure and morphology of the bulk. The NCA/CNT composite exhibits enhanced cycling and rate performance compared with pristine NCA. After 60 cycles at a current rate of 0.25 C, the reversible capacity of NCA/CNT composite cathode is 181 mAh/g with a discharge retention rate of 96%, considerably higher than the value of pristine NCA (153 mAh/g with a retention rate of 90%). At a high current rate of 5 C, it also can deliver a reversible capacity of 160 mAh/g, while only 140 mAh/g is maintained for the unmodified NCA. Highly electrical conductive CNTs rather than common inert insulating materials are for the first time employed as surface modifiers for NCA, which are dispersed homogenously on the surface of NCA particles, not only improving the electrical conductivity but also providing effective protection to the side reactions with liquid electrolyte of the battery.

## Background

Due to its excellent cyclability and high energy density, lithium-ion batteries (LIBs) are playing a crucial role in the modern society. Typically, anode materials of LIBs are of low cost, offering relatively high capacity, while cathode materials are facing drawbacks of lower capacity and higher cost. Therefore, pursuit of LIB cathode materials with higher energy density is of great importance and demanding [1–3].

Along with the development of cathode materials for LIBs, lithium storage properties of hexagonal layer structured LiCoO_2_ (theoretical specific capacity 274 mAh/g) has been thoroughly studied. During charge-discharge process, LiCoO_2_ shows excellent reversible capacity (usually ~150 mAh/g) and remarkable cycling stability [4, 5]. However, due to the toxicity and high cost of cobalt metal, layered nickel oxides (e.g., LiNiO_2_) have been developed as alternatives for cathode, providing 10–30 mAh/g higher specific capacity than LiCoO_2_ in real practice despite their same theoretical capacity, but unstable highly oxidized Ni^4+^ ions are generated upon delithiation, resulting in side reactions with electrolyte, hence poor cycling and thermal stability of the batteries. In addition, synthesizing LiNiO_2_ at accurate stoichiometry is challenging, which also hinders the commercial application of LiNiO_2_ [6, 7]. However, it was found that partial replacement of Ni^3+^ with Co^3+^ at the same location in LiNiO_2_, i.e., LiNi_1−*x*_Co_*x*_O_2_, could significantly increase the capacity as well as the cycling stability [8, 9].

Furthermore, ternary cathode material LiNi_1−*x*−*y*_Co_*x*_Al_*y*_O_2_ was fabricated by co-substituting Ni^3+^ with Al^3+^ and Co^3+^ in the LiNiO_2_ compound [10]. Such cathode materials have advantages of improved electrochemical properties and thermal stability, low cost, and low toxicity. Among the diverse Ni-based ternary layered metal oxide materials, LiNi_0.8_Co_0.15_Al_0.05_O_2_ (*x* = 0.15, *y* = 0.05) attracts most attention when applied to LIBs due to the optimal balance between capacity and structural stability. Therefore, we refer NCA in this article specifically to LiNi_0.8_Co_0.15_Al_0.05_O_2_. Nevertheless, there remain problems unsolved: (1) Residual Ni^2+^ in NCA tends to migrate from transition metal layers to the Li^+^ slabs and form electrochemically inactive NiO-like phase, resulting degradation of cathode during charge-discharge process; (2) Side reactions of highly oxidized Ni^4+^ with electrolyte during cycling is another main reason responsible for the degradation of NCA; (3) Moreover, poor electrical conductivity of the pristine material also impairs its electrochemical performance [11, 12]. Consequently, improvement on the cycling stability and safety is of primary concern in the research on NCA.

As degradation generally starts from the surface of the NCA particles, surface modification has been widely adopted as an efficient method to prevent/suppress side reactions with the electrolyte for the purpose of improved cycling stability, rate capability, and thermal stability [13]. The most commonly used modification strategy is through chemical coating a uniform nanoscale protective layer of TiO_2_ [14], MnO_2_ [15], ZrO_2_ [16], FePO_4_ [17], or AlF_3_ [18], etc. onto the NCA particle surface, following a process of solvent evaporation and high temperature annealing. Such wet-coating method is effective, however, requires additional post-treatment, which is time and energy consuming. On the other hand, mechanical ball-milling composites of NCA and nanoparticles such as SiO_2_ [19], Ni_3_ (PO_4_)_2_ [20], and AlF_3_ [21] have also shown remarkably improved electrochemical performance. The mechanical mixing process is relatively simple, clean, low cost and poses less side effect on ion/electron transference compared to full coating an insulating layer via chemical route. But stringent control of milling speed and time is critical in order to realize homogenous dispersion of the modifying nanoparticles and at the same time remains the integration of the NCA particles. Moreover, to our best knowledge, except one NCA/graphene composite cathode prepared by ball-milling [22], almost all the reported modifiers so far are inert materials, which although showing good stability have poor electrical conductivity associated with increased polarization of the electrode materials.

In this study, for the first time, carbon nanotubes (CNTs) are employed as the surface modifier for NCA by a simple mechanical grinding method. On the one hand, gentle grinding rather than vigorous ball-milling can avoid damage to material crystal structure and morphology; on the other hand, CNTs, which can be well dispersed on the NCA particles surface, provide the electrode better electrical conductivity and effective protection. Therefore, NCA/CNT composite cathode exhibits enhanced specific capacity and rate capability. The structure, morphology, and electrochemical properties have been analyzed in details.

## Methods

Both NCA and CNTs were commercially supplied. To prepare NCA/CNT composite, pristine NCA was first ground with 5, 10, and 20 wt% of the CNTs using a pestle and agate mortar at room temperature for 1 h. The microstructure and morphology were observed by field emission scanning electron microscopy (FESEM, Quanta FEI, America). Powder X-Ray diffraction (XRD) patterns were recorded on a Rigaku (Smart Lab III) using Cu Kα radiation within 2θ = 10–80° with a step width of 0.05°. Raman spectroscopy measurements were performed on a laser Raman spectrometer (LabRAM HR, France) with a He-Ne laser (532 nm) as the excitation source. Energy dispersive X-ray spectrometry (EDS) was also applied to identify the distribution of elements in the composite.

The working electrodes were fabricated from slurries of the active materials (80 wt%), acetylene black (10 wt%), and polyvinylidene fluoride (10 wt%) mixed in the solvent *N*-methyl-2-pyrrolidone (NMP). The slurries were then cast onto an aluminum foil and dried at 100 °C in vacuum overnight. Electrochemical characterizations were performed on a CR2032 coin-type cell with lithium metal as the counter electrode and 1M LiPF_6_ in an ethylene carbonate/dimethyl carbonate (1:1 in volume) solution as the electrolyte. The cells were assembled in an argon-filled glove box. Galvanostatic charge/discharge measurements were carried out between 2.8 and 4.3 V (vs. Li/Li^+^) using a battery test system LAND CT2001A. Cyclic voltammetry (CV) was carried out in the potential range of 2.8–4.5 V (vs. Li/Li^+^) with a scan rate of 0.1 mV/s. Ac impedance spectroscopy (EIS) was measured by applying an Ac voltage of 5 mV in the frequency range of 100 kHz to 0.01 Hz using Biologic VMP3 electrochemical workstation.

## Results and Discussion

**Fig. 1:**
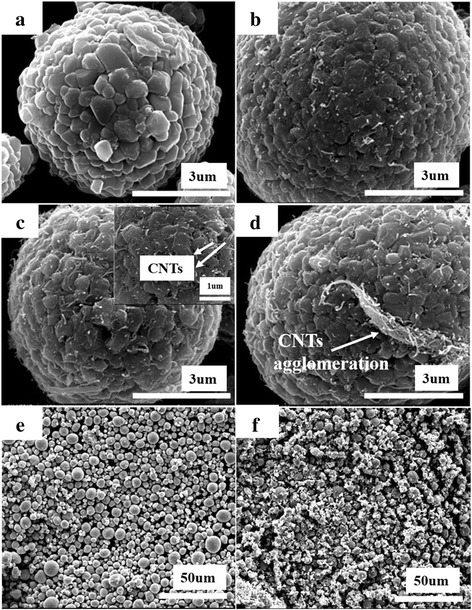
SEM images of **a** pristine NCA and **b** 5 wt% CNT, **c** 10 wt% CNT, **d** 20 wt% CNT-composited NCA. SEM images of pristine NCA **e** ground in agate mortar for 1 h and **f** ball-milled at 100 rpm for 1 h

Figure 1a–d are the SEM images of pristine NCA and NCA/CNT composites with different content of CNTs. As depicted in Fig. 1a, the pristine NCA is composed of secondary microspheres with a diameter range of 5–8 μm containing numerous primary nanoparticles with particle sizes of 100 to 500 nm. This also explains that over strong mechanical forces such as high energy ball-milling may crush the secondary structures of NCA, influencing its electrochemical properties. Such speculation is further confirmed by Fig. 1e, f, the SEM images of pristine NCA ground at agate mortar for 1 h and ball-milled at a rotation speed of 100 rpm for 1 h, respectively. NCA particles remain intact after grinding, while agglomeration of broken NCA pieces is clearly observed in the ball-milled analogue. Fig. 1b–d compare the morphology of NCA/CNT composites varying the CNT content. As we can see, with increasing CNTs, more CNTs are attracted to the surface of NCA particles. However, extra accumulation of CNTs takes place when its content increases to 20 wt%. As shown in the inset of Fig. 1c, one also can clearly see that CNTs adhere tightly and homogeneously to the surface of the NCA particles. Therefore, in the discussion below, we will focus on the NCA/CNT composite mechanically mixed with 10 wt% CNTs.

**Fig. 2:**
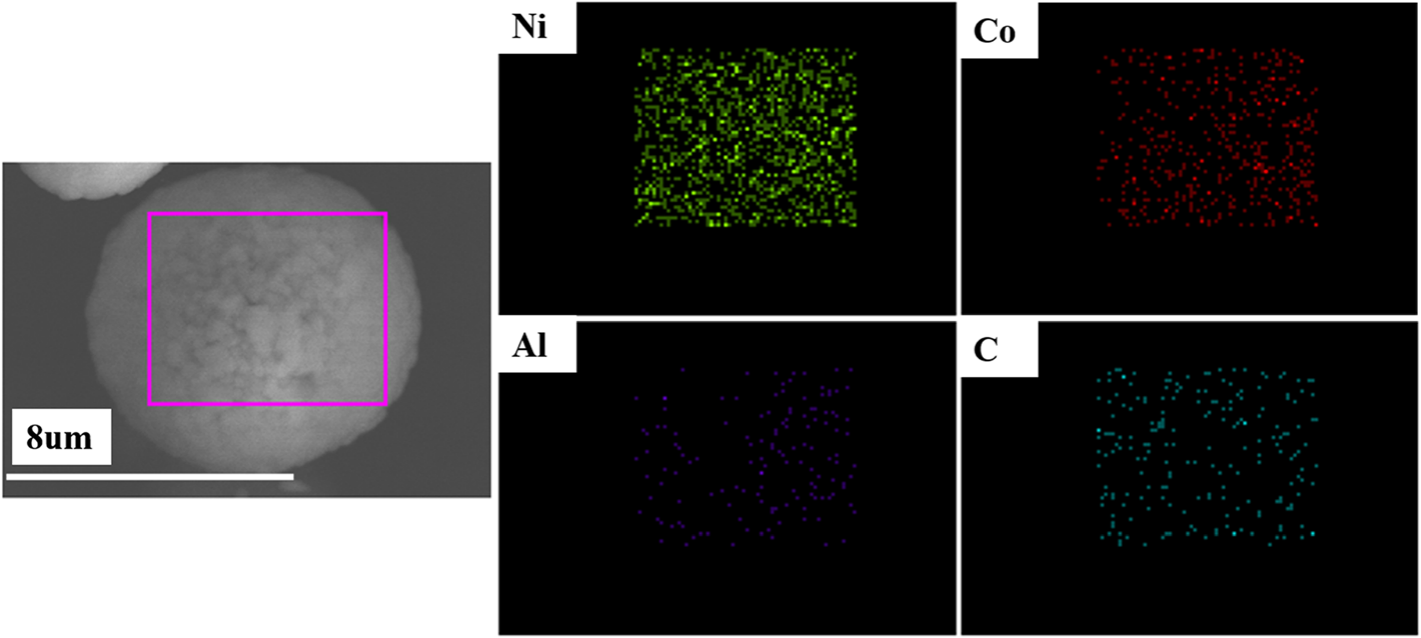
EDS dot-mapping images of Ni, Co, Al, and C elements of NCA/CNT (10 wt%) composite

Figure 2 shows the EDS dot-mapping images of Ni, Co, Al, and C elements in the NCA/CNT composite, which reveals that C element, similar to other elements (Ni, Co, Al) associated with NCA, homogenously distributes in the selected region of the composite microsphere.

**Fig. 3:**
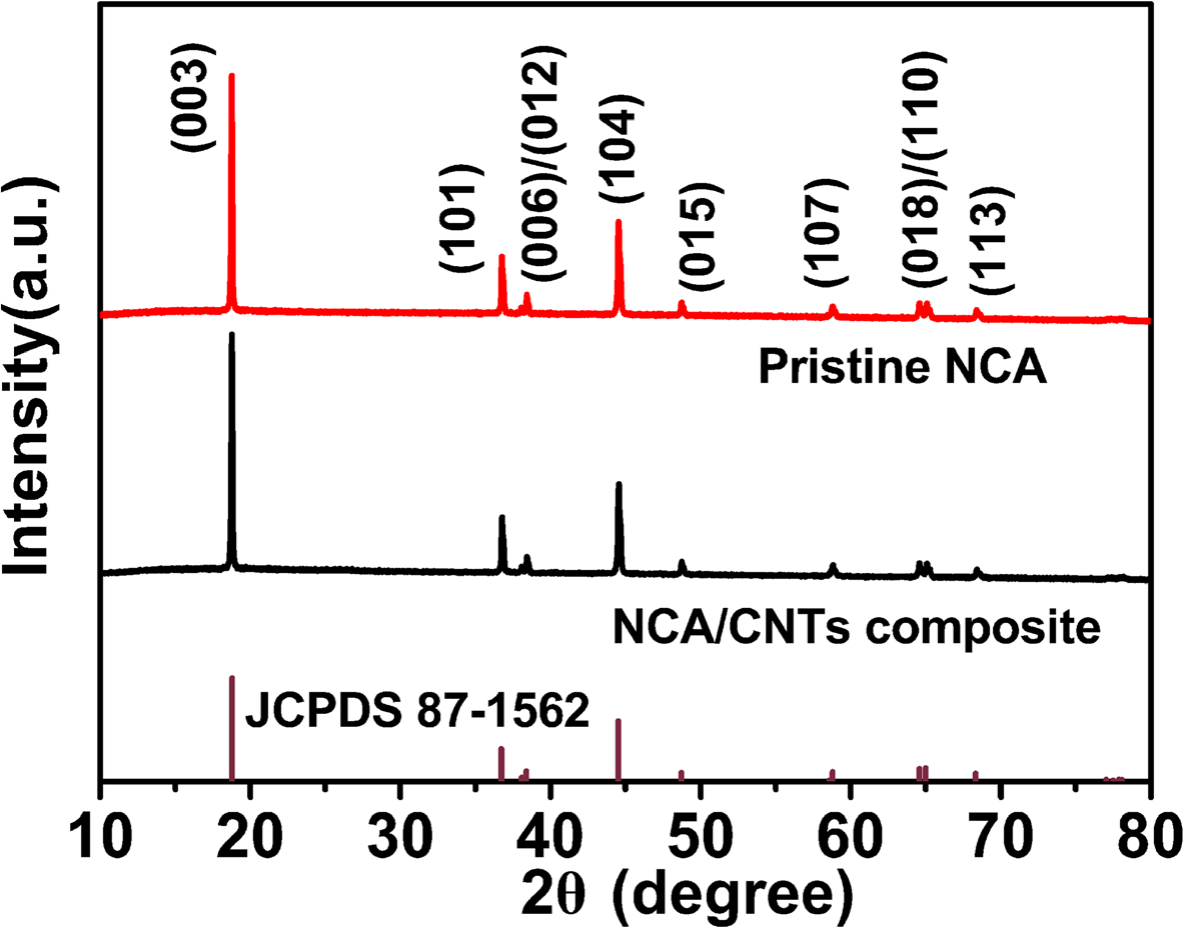
XRD patterns of pristine NCA and NCA/CNT (10 wt%) composite

Figure 3 shows the X-ray diffraction (XRD) patterns of the pristine and CNT-composited NCA material. All the diffraction peaks of both samples can be indexed to a typical hexagonal α-NaFeO_2_ layered structure with R3m spacing group. The (003) peak centered at 2θ = 18.73° and (104) peak centered at 2θ = 44.52° correspond to the reflection of R3m layered rock salt structure and the mixed reflections of R3m layered rock salt structure and Fm3m cubic rock salt structure, respectively [23–25]. Neither characteristic peak of CNTs (2θ = 25°) nor other impurity peaks are detected in the XRD pattern of the composite, indicating that NCA is highly crystalized and its crystal structure is unaffected by the grinding method.

**Fig. 4:**
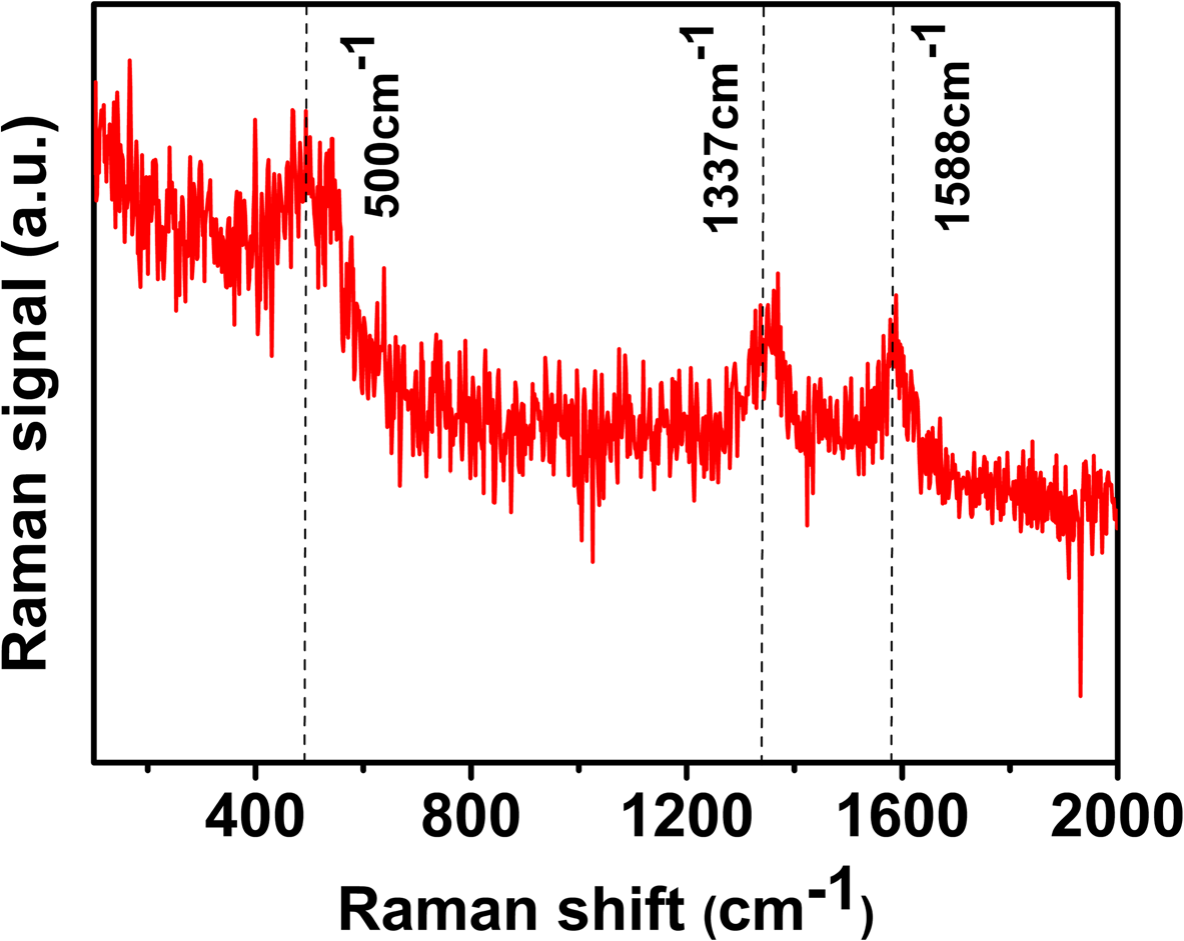
Raman spectrum of NCA/CNT (10 wt%) composite

The Raman spectrum of NCA/CNT composite is shown in Fig. 4. The broad Raman band at ~500 cm^−1^ is assigned to the vibrational bending (*E*_*g*_) and stretching (*A*_*1g*_) modes in NCA [26]. The composite presents a prominent G-band (graphite carbon band) at 1588 cm^−1^ corresponding to the in-plane vibration of sp2 carbon atoms, as well as a D-band (disordered carbon band) at 1337 cm^-1^ [27, 28], confirming the existence of CNTs.

**Fig. 5:**
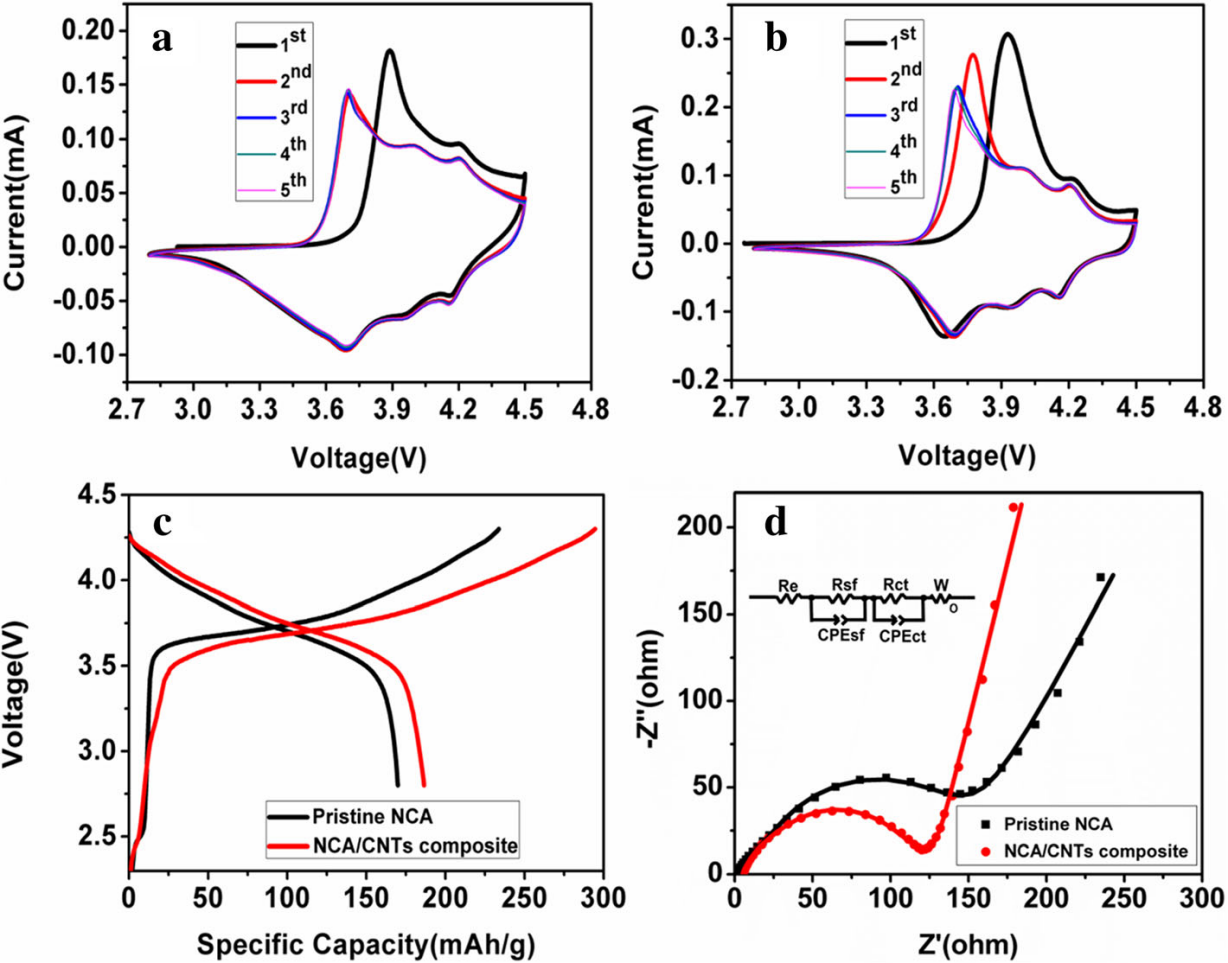
Cyclic voltammograms of **a** pristine NCA and **b** NCA/CNT (10 wt%) composite. **c** The initial charge-discharge curves at 0.25 C rate and **d** Nyquist plots (*inset*: equivalent circuit used to fit the experimental data) of pristine NCA and NCA/CNT (10 wt%) composite

Figure 5a, b display the cyclic voltammetry (CV) curves of the pristine NCA and NCA/CNT composite, respectively. As shown in Fig. 5a, for pristine NCA, two oxidative peaks at 3.9 and 4.2 V are presented in the first cycle, while from the second cycle, the strong oxidative peak at 3.9 V shifts to a lower potential (3.75 V) and three redox pairs at 3.75 V/3.7 V, 4.0 V/3.96 V and 4.2 V/4.18 V appear, which are attributed to phase transitions of hexagonal (H1) to monoclinic (M), monoclinic to hexagonal (H2), and hexagonal (H2) to hexagonal (H3) during the Li^+^ extraction/insertion in NCA [29–31]. The CV profiles of NCA/CNT composite electrode are very similar to those of pristine NCA, except that irreversible phase change still occurs in the second cycle, indicating a slower structural dynamics due to the presence of CNTs (Fig. 5b). From the third cycle onward, the cathodic and anodic peaks reproduce very well, showing stable cycling performance of the composite cathode.

The initial charge-discharge profiles of pristine NCA and NCA/CNT composite under a current rate of 0.25 C (1C = 200 mA/g), between 2.8 and 4.3 V, are illustrated in Fig. 5c. Both cathodes show a typical plateau characteristic of NCA material at around 3.7 V. However, a slightly lower charge plateau and higher discharge plateau are obvious for NCA/CNT composite, indicating a smaller polarization of the electrode benefiting from addition of the highly conductive CNTs. The better conductivity of NCA/CNT composite electrode is further confirmed using Ac impedance spectroscopy (Fig. 5d). Two overlapped depressed semi-circles at high frequency along with an inclined spike at low frequency are observed for both spectra. The two semi-circles represent the solid electrolyte interphase (SEI) impedance and the charge-transfer impedance at the electrode/electrolyte interface respectively, whereas the straight line is associated with diffusion of Li^+^ through electrode material [32]. An equivalent circuit has been used to quantify the influence of CNTs on the Li^+^ transport (inset of Fig. 5d), in which R_e_ represents the electrolyte resistance and R_sf_, R_ct_, CPE_sf_, and CPE_ct_ are the resistances and capacitances of SEI film and interface, respectively, and Z_W_ is the Warburg impedance. As can be seen, the total resistance (R_e_ + R_s_ + R_ct_) of the NCA/CNT composite (110.83 Ω) is significantly smaller than that of the pristine NCA (145.13 Ω).

In addition, the initial charge and discharge specific capacities of NCA/CNT composite are 295 and 187 mAh/g, respectively, which are remarkably higher than those of the pristine NCA (234 mAh/g, 170 mAh/g). One should note that NCA/CNT composite has lower initial coulombic efficiency (63%) than pristine NCA (72%), which might be ascribed to the irreversible phase change and formation of more SEI film companying with the presence of high surface area CNTs.

**Fig. 6:**
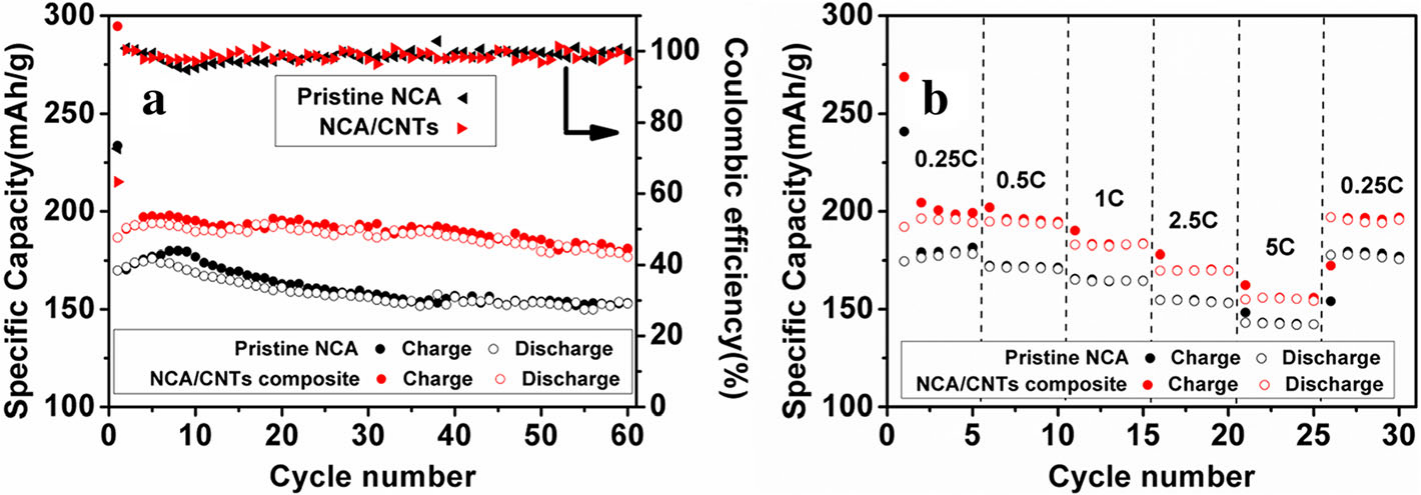
**a** Cycling performance at 0.25 C rate. **b** Rate performance of pristine NCA and NCA/CNT (10 wt%) composite

Figure 6a compares cycling performance between the pristine NCA and NCA/CNT composite at 0.25 C rate. The capacity fading is apparently less pronounced for the composite. After 60 cycles, the composite can remain a reversible specific capacity as high as 181 mAh/g, while the pristine NCA only shows 153 mAh/g. From the second cycle, the coulombic efficiency of NCA/CNT composite can retain above 99%. The rate capability of the NCA/CNT composite is also greatly enhanced compared to pristine NCA as shown in Fig. 6b. It is clear to see that NCA/CNT composite exhibits much higher stable capacity at each current rate (from 0.25 to 5 C) than the pristine analogue, and at high current rate of 5 C, it still delivers a charge/discharge capacity of 160 mAh/g, while NCA drops to 140 mAh/g. When the current density restores to initial 0.25 C, nearly 100% charge-discharge specific capacity of NCA/CNT composite can be restored, demonstrating an excellent reversibility.

## Conclusions

In this paper, NCA/CNT composite cathode materials are prepared by a simple mechanical solid-state grinding method without damage to the crystal structure and morphology of raw NCA material. The highly conductive CNTs are dispersed homogeneously on the surface of NCA particles. Presence of CNTs not only offers the electrode a better electrical conductivity but also effectively suppresses side reactions of NCA particles with liquid electrolyte. The cycling performance and rate capability are therefore greatly improved compared to pristine NCA. After 60 cycles at 0.25 C rate, the reversible specific capacity of NCA/CNT composite is 181 mAh/g, enhanced by 18% than pristine NCA (153 mAh/g). At high current rate of 5 C, NCA/CNT composite still can deliver a reversible specific capacity as high as 160 mAh/g, while pristine NCA only has 140 mAh/g.

## Abbreviations

CNTs: Carbon nanotubes
LIB: Lithium-ion battery
NCA: LiNi_0.8_Co_0.15_Al_0.05_O_2_
